# The Exploitation of Host 26S Proteasome as a New Stratedy for Bacterial Pathogenicity

**DOI:** 10.3389/fpls.2022.858829

**Published:** 2022-03-31

**Authors:** Chaofeng Wang, Lirong Zeng

**Affiliations:** Department of Plant Pathology, Center for Plant Science Innovation, University of Nebraska, Lincoln, NE, United States

**Keywords:** proteasome, ubiquitin-independent protein degradation, bacterial pathogenicity, vector borne disease, phytoplasma, effector, RPN10

The 26S proteasome serves as a key hub for modulating host immune responses by the ubiquitin-proteasome system (UPS). Huang and colleagues revealed that a phytoplasma effector targets a plant's 26S proteasome to promote colonization through ubiquitin-independent degradation. Blocking this degradation step confers the *Arabidopsis thaliana* plants' resistance to this potent effector.

Phytoplasmas are cell wall-absent and phloem-restricted obligate bacterial pathogens. Transmission of phytoplasmas is mediated by phloem-feeding insects, including leafhoppers, planthoppers, and psyllids. In their dual-host life cycle, the phytoplasmas are often neutral or beneficial to their insect vectors, yet detrimental to hundreds of plant species globally, including many economically important crops (Malembic-Maher et al., 2020). Infection by phytoplasmas often induces substantial architectural changes in plants, such as witches' broom (clustering of branches with tiny leaves) and phyllody (retrograde development of the floral organs). Phytoplasma-infected plants have been dubbed “zombie plants” because they grow slower than normal plants with a reproduction stoppage, which serves as favorite habitat for their insect vectors. The phenomenon that phytoplasmas turn their host plants into zombies is known as “extended phenotype,” which was first coined by the British evolutionary biologist Richard Dawkins in 1982. Despite tremendous efforts, the molecular mechanisms underlying the modulation of host plants into “zombie” by phytoplasmas remain poorly understood.

To address this question, Huang et al. focused on an effector protein, the SAP05 from the Aster Yellows phytoplasma (AYP) strain Witches' Broom (AY-WB; *Candidatus* Phytoplasma *asteris*), that can infect the model plant *Arabidopsis thaliana* (Huang et al., 2021). They concentrated on the SAP05 because the *Arabidopsis'* transgenic lines over-expressing SAP05 exhibited typical witches' broom symptoms and significant loss of fertility. The transgenic plants also lacked serrated edges and delayed abaxial trichomes appearance on matured rosette leaves, suggesting that SAP05 likely disturbs vegetative phase transition, a development process that is regulated by the miR156-SQUAMOSA PROMOTER BINDING PROTEIN-LIKE (SPL) transcription factor (TF) module (Zheng et al., 2019) (Figure 1A). For consistency, the authors revealed *via* yeast two-hybrid (Y2H) screens that SAP05 interacts with several SPL and GATA TFs that are vital to the plant's developmental phase transitions and flower organ development, respectively (Ranftl et al., 2016; Zheng et al., 2019) (Figure 1A). The *Arabidopsis* RPN10 protein, a major receptor within the 19S regulatory particle (RP) of the 26S proteasome, was also identified in the Y2H screen. The RPN10 recognizes and captures ubiquitinated substrates through its C-terminal ubiquitin-interacting motifs (UIM) domain, whereas its N-terminal vWA (von Willebrand factor A) domain is required for the proteasome association (Smalle et al., 2003).

**Figure 1:**
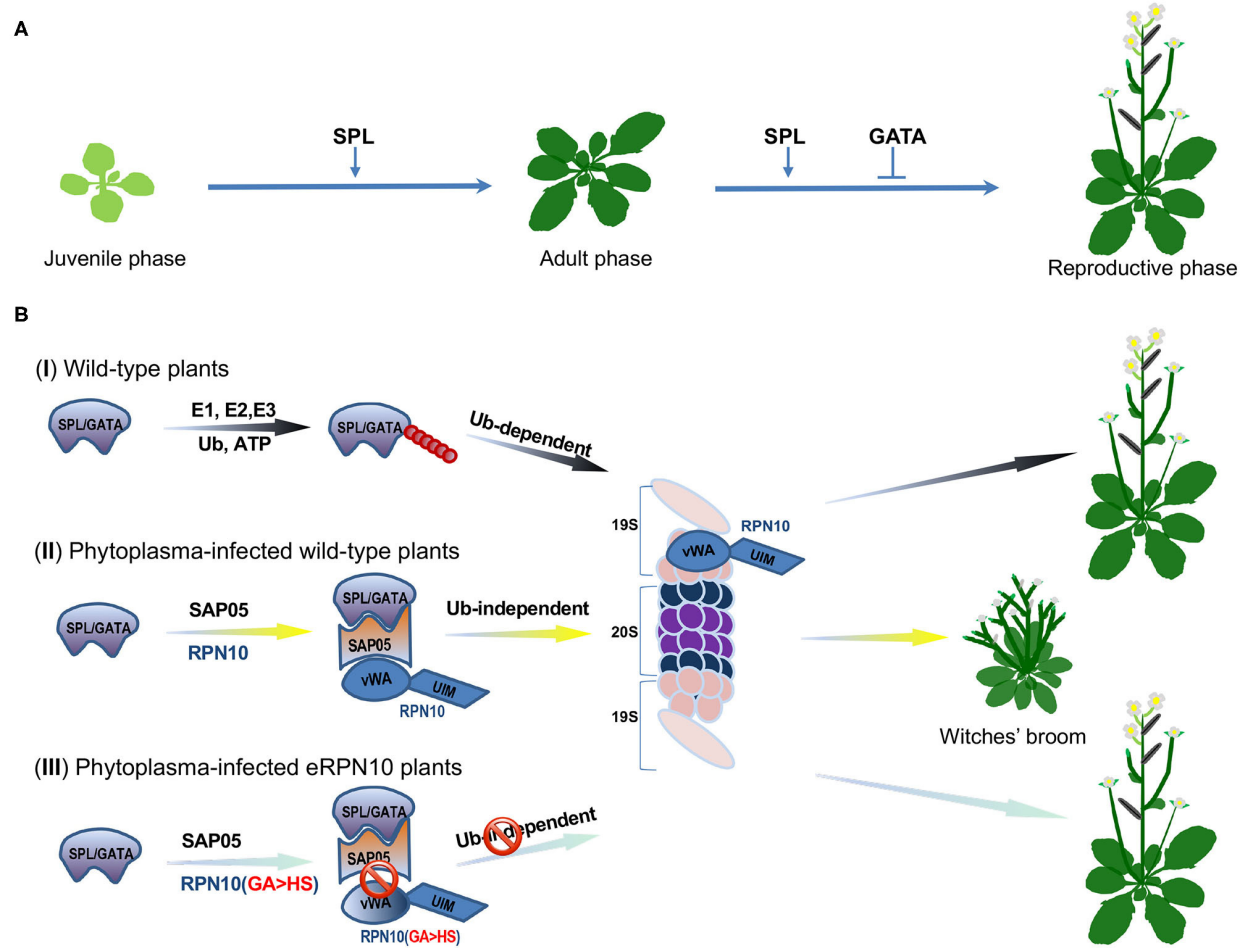
SAP05 modulates host plant developmental processes by hijacking the RPN10 subunit of plant 26S proteasome. **(A)** A schematic model of Squamosa promoter binding protein-like (SPL) and GATA transcription factors (TFs)-orchestrated wild-type plant growth and development. The SPLs promote the juvenile-to-adult and adult-to-reproductive phase transition, whereas GATA proteins suppress floral organ development. **(B)** Diagrams of SPL/GATA TFs degradation at the plant 26S proteasome. (I) Under normal growth conditions, SPL/GATA TFs are constitutively subjected to ubiquitin-dependent degradation by the ubiquitin-proteasome system (UPS) to meet the demands of plant growth and development. The ubiquitin molecules are depicted as filled red circles. (II) In phytoplasma-infected plants, the effector SAP05 forms a bridge between host ubiquitin receptor RPN10 and SPLs/GATAs to mediate ubiquitin-independent degradation of these TFs at the 26S proteasome. The degradation of SPL and GATA proteins leads to the decoupling of developmental phase transitions and, thus, keeps the host plant stay at the juvenile stage with more lateral shoots, secondary branches, and sterile flowers. (III) The SAP05 fails to bind the engineered plant RPN10 with two amino acids (38GA39) being replaced with HS (eRPN10 or RPN10 m1). The disassociation of SAP05 and eRPN10 abolishes the ubiquitin-independent degradation of SPL/GATA TFs, conferring the eRPN10-expressing plant's resistance to SAP05 activities, thus, maintaining wild-type architecture. The SPL/GATA proteins are represented as a light purple irregular shape, the SAP05 is delineated in orange, the RPN10 and eRPN10 are rendered in blue, the subunits of the 19S regulatory particle of the 26S proteasome are depicted in light salmon, whereas the α and β subunits that constitute the 20S proteasome complex are in dark blue and purple, respectively.

These findings prompted Huang et al. to investigate if SAP05 can mediate the degradation of the SPLs and GATAs. Degradation assays, using wild-type and *rpn10-2 Arabidopsis* mesophyll protoplasts and in *Nicotiana benthamiana* (*Nb*) leaves, indicated that co-expression of SAP05 with SPL/GATA led to a reduced or abolished accumulation of these TFs in an RPN10-dependent manner. The examination of the interactions among SPL/GATA, SAP05, and RPN10 suggested that SAP05 acts as a bridge between SPLs/GATAs, and RPN10 forms a ternary complex that facilitates degradation of SPLs/GATAs at the 26S proteasome (Figure 1B). Interestingly, the SAP05 interacts with the vWA domain but not with the UIM domain of RPN10, raising the question of whether ubiquitination is required for SAP05-mediated degradation of SPLs and GATAs. The authors found that SAP05 can still promote degradation of AtGATA18 and AtGATA19, even all lysines in the two proteins were replaced with arginine. In a separate assay, the authors found a co-expression of SAP05 with GATA19, and the SPL5 in *Arabidopsis* protoplasts in the presence of dominant-negative ubiquitin (Ub-ΔGG) that blocks conjugation of ubiquitin chain to substrate proteins still resulted in degradation of GATA19 and SPL5. The authors also found that SPL5 can be degraded by purified human 26S proteasome *in vitro* when AtPRN10 vWA and SAP05 were presented in the assay where enzymes required for ubiquitination were absent. These overall results indicated that ubiquitination is not required for SAP05-mediated SPL/GATA degradation.

The Aster Yellows phytoplasma strain witches' broom (AY-WB) causes diseases on the *A. thaliana* plants but does not usually interfere with their insect vectors. Thus, a relevant question that arose from the above findings was whether SAP05 interacts with the insect RPN10 protein. The authors found no interactions between SAP05 and MqRPN10, the AtRPN10 homolog in leafhopper. The sequence alignment, based on the vWA domain of plant and animal RPN10 homologs, revealed two major differences: the amino acids 38–39 are GA in plants but HS in animals, and amino acids 56–58 are GKG in plants vs. K– in animals. Huang et al. then created two AtRPN10 variants: AtRPN10_38GA39 to HS (AtRPN10 m1) and AtRPN10_56GKG58 to K (AtRPN10 m2) (Figure 1B). The ensued Y2H assay revealed that the two mutants did not interact with SAP05. When either of the two mutants was used to complement the *rpn10-2* protoplasts in the presence of SAP05, the degradation of GATA18 and SPL5 was diminished. Notably, the reduction in GATA18 and SPL5 degradation was more significant in the presence of AtRPN10 m1 compared with AtRPN10 m2, which suggests that 38GA39 of AtRPN10 plays a major role in SAP05's binding and activities. The authors further showed that *rpn10-2* plants complemented with the AtRPN10 m1 (eRPN10, for engineered RPN10) displayed resistance to phytoplasmas SAP05-induced developmental changes in *A. thaliana*, which raises the possibility of generating phytoplasma-resistant plants by engineering plant RPN10. However, the transgenic plants expressing the eRPN10 still displayed symptoms induced by other effectors, such as phyllody mediated by the AY-WB effector SAP54 (MacLean et al., 2014). Thus, the generation of plants with host targets of multiple effectors being modified simultaneously may be a more effective way to engineer plant resistance to the AY-WB phytoplasma.

During the lengthy coevolution with their hosts, many microbial parasites and pathogens have developed strategies to manipulate various host's cellular and physiological processes for their advantage. Increasing evidence indicates that components of the host's UPS are the attractive target of such manipulation because UPS plays critical roles in a broad array of cellular responses, thus, targeting it can have a broader impact on the host. The 26S proteasome serves as a central hub for protein degradation by UPS (Langin et al., 2020). Though several bacterial effectors have been shown to inhibit host proteasome activity as a virulence strategy (Ramachandran et al., 2021), the hijack of the 26S proteasome for degradation of host proteins to promote virulence has rarely been reported. Huang et al. revealed in this new study that the SAP05 effector of phytoplasmas utilizes the plant 26S proteasome to degrade multiple host proteins in a ubiquitin-independent manner, which explains why SAP05 alone can induce dramatic developmental changes to produce the witches' broom symptoms. This new strategy employed by SAP05 for protein degradation apparently avoids the steps required for ubiquitination and subsequent ubiquitin recycling, thus, reducing the chance of being detected by the plant's immune surveillance system. Studies on a limited number of proteins, known to be subject to ubiquitin-independent degradation, indicate that proteasome association is a prerequisite for this degradation pathway (Erales and Coffino, 2014). Similarly, the phytoplasma have evolved SAP05 to facilitate the association of SPLs/GATAs with the proteasome yet avoiding interference of the counterpart of the insect vector. In this regard, the SPA05 is likely to be a product of the coevolution of phytoplasmas, with both the host plants and the insect vectors.

The underpinning molecular mechanisms for ubiquitin-independent degradation at the proteasome remain poorly understood, thus far. By deciphering the molecular basis underlying SAP05-mediated virulence, Huang and colleagues elucidated a novel mechanism for ubiquitin-independent proteolysis at the proteasome. Through engineering the RPN10 subunit of the 26S proteasome, the authors also presented a new strategy for the control of vector-borne diseases. Considering the great challenges of vector-borne diseases and that we still lack a cure for many of them, the significance of this study can hardly be overstated.

## Author Contributions

CW wrote draft of the manuscript. LZ edited and finalized the manuscript. All the authors contributed to the article and approved the submitted version.

## Funding

CW was supported by a National Science Foundation Grant (IOS-1645659) to LZ.

## Conflict of Interest

The authors declare that the research was conducted in the absence of any commercial or financial relationships that could be construed as a potential conflict of interest.

## Publisher's Note

All claims expressed in this article are solely those of the authors and do not necessarily represent those of their affiliated organizations, or those of the publisher, the editors and the reviewers. Any product that may be evaluated in this article, or claim that may be made by its manufacturer, is not guaranteed or endorsed by the publisher.

## References

[B1] Erales J. Coffino P. (2014). Ubiquitin-independent proteasomal degradation. Biochim. Biophys. Acta. 1843, 216–221. 10.1016/j.bbamcr.2013.05.008 23684952 PMC3770795

[B2] Huang W. MacLean A. M. Sugio A. Maqbool A. Busscher M. Cho S. T.. (2021). Parasitic modulation of host development by ubiquitin-independent protein degradation. Cell 184, 5201–5214. 10.1016/j.cell.2021.08.029 34536345 PMC8525514

[B3] Langin G. Gouguet P. Üstün S. (2020). Microbial effector proteins - a journey through the proteolytic landscape. Trends Microbiol. 28, 523–535. 10.1016/j.tim.2020.02.010 32544439

[B4] MacLean A. M. Orlovskis Z. Kowitwanich K. Zdziarska A. M. Angenent G. C. Immink R. G.. (2014). Phytoplasma effector SAP54 hijacks plant reproduction by degrading MADS-box proteins and promotes insect colonization in a RAD23-dependent manner. PLoS Biol 12, e1001835. 10.1371/journal.pbio.1001835 24714165 PMC3979655

[B5] Malembic-Maher S. Desque D. Khalil D. Salar P. Bergey B. Danet J. L.. (2020). When a Palearctic bacterium meets a Nearctic insect vector: genetic and ecological insights into the emergence of the grapevine Flavescence doree epidemics in Europe. PLoS Pathog. 16, e1007967. 10.1371/journal.ppat.1007967 32210479 PMC7135369

[B6] Ramachandran P. J B. Maupin-Furlow J. Uthandi S. (2021). Bacterial effectors mimicking ubiquitin-proteasome pathway tweak plant immunity. Microbiol. Res. 250, 1268 10.1016/j.micres.2021.126810 34246833 PMC9523578

[B7] Ranftl Q. L. Bastakis E. Klermund C. Schwechheimer C. (2016). LLM-Domain containing B-gata factors control different aspects of cytokinin-regulated development in Arabidopsis thaliana. Plant Physiol. 170, 2295–2311. 10.1104/pp.15.01556 26829982 PMC4825128

[B8] Smalle J. Kurepa J. Yang P. Emborg T. J. Babiychuk E. Kushnir S.. (2003). The pleiotropic role of the 26S proteasome subunit RPN10 in Arabidopsis growth and development supports a substrate-specific function in abscisic acid signaling. Plant Cell 15, 965–980. 10.1105/tpc.009217 12671091 PMC152342

[B9] Zheng C. Ye M. Sang M. Wu R. (2019). A regulatory network for miR156-SPL module in Arabidopsis thaliana. Int. J. Mol. Sci. 20, 6166. 10.3390/ijms20246166 31817723 PMC6940959

